# Hippocampus, Amygdala, and Thalamus Volumes in Very Preterm Children at 8 Years: Neonatal Pain and Genetic Variation

**DOI:** 10.3389/fnbeh.2019.00051

**Published:** 2019-03-19

**Authors:** Cecil M. Y. Chau, Manon Ranger, Mark Bichin, Min Tae M. Park, Robert S. C. Amaral, Mallar Chakravarty, Kenneth Poskitt, Anne R. Synnes, Steven P. Miller, Ruth E. Grunau

**Affiliations:** ^1^Department of Pediatrics, The University of British Columbia, Vancouver, BC, Canada; ^2^BC Children’s Hospital Research Institute, Vancouver, BC, Canada; ^3^School of Nursing, The University of British Columbia, Vancouver, BC, Canada; ^4^Department of Psychiatry, The University of Western Ontario, London, ON, Canada; ^5^Cerebral Imaging Centre, Douglas Mental Health University Institute, Montreal, QC, Canada; ^6^Department of Paediatrics, The Hospital for Sick Children, University of Toronto, Toronto, ON, Canada

**Keywords:** preterm, pain, stress, hippocampus, amygdala, thalamus, limbic, genes

## Abstract

Altered hippocampal morphology and reduced volumes have been found in children born preterm compared to full-term. Stress inhibits neurogenesis in the hippocampus, and neonatal stress/noxious stimulation in rodent pups are associated with long-term alterations in hippocampal volumes. We have previously shown reduced cortical thickness and cerebellar volumes in relation to more exposure to pain-related stress of neonatal invasive procedures in children born very preterm. We have reported targeted gene-by-pain environment interactions that contribute to long-term brain development and outcomes in this population. We now aim to determine whether exposure to pain-related stress (adjusted for clinical factors and genotype) differentially impacts regional structures within the limbic system and thalamus, and investigate relationships with outcomes in very preterm children. Our study included 57 children born very preterm (<32 weeks GA) followed longitudinally from birth who underwent 3-D T1 MRI neuroimaging at ∼8 years. Hippocampal subfields and white matter tracts, thalamus and amygdala were automatically segmented using the MAGeT Brain algorithm. The relationship between those subcortical brain volumes (adjusted for total brain volume) and neonatal invasive procedures, gestational age (GA), illness severity, postnatal infection, days of mechanical ventilation, number of surgeries, morphine exposure, and genotype (*COMT, SLC6A4*, and *BDNF*) was examined using constrained principal component analysis. We found that neonatal clinical factors and genotypes accounted for 46% of the overall variance in volumes of hippocampal subregions, tracts, basal ganglia, thalamus and amygdala. After controlling for clinical risk factors and total brain volume, greater neonatal invasive procedures was associated with lower volumes in the amygdala and thalamus (*p* = 0.0001) and an interaction with *COMT* genotype predicted smaller hippocampal subregional volume (*p* = 0.0001). More surgeries, days of ventilation, and lower GA were also related to smaller volumes in various subcortical regions (*p* < 0.002). These reduced volumes were in turn differentially related to poorer cognitive, visual-motor and behavioral outcomes. Our findings highlight the complexity that interplays when examining how exposure to early-life stress may impact brain development both at the structural and functional level, and provide new insight on possible novel avenues of research to discover brain-protective treatments to improve the care of children born preterm.

## Introduction

Children born very preterm [24–32 weeks gestational age (GA)] are at risk for cognitive, emotional and behavior problems, persisting to adulthood (e.g., Anderson et al., 2003; Grunau et al., 2004; Doyle and Anderson, 2010; Linsell et al., 2018). Very preterm infants are exposed to developmentally unexpected environmental stress in the neonatal intensive care unit (NICU) during a critical period of rapid brain development and programming of stress systems. These infants undergo about 10 noxious procedures per day (Simons et al., 2003; Carbajal et al., 2008; Johnston et al., 2011; Roofthooft et al., 2014). In infants born very preterm, we have previously reported that greater exposure to neonatal pain-related stress is associated with altered brain microstructure (Brummelte et al., 2012; Ranger et al., 2013, 2015; Zwicker et al., 2013; Vinall et al., 2014) and processing (Doesburg et al., 2013), as well as programming of the hypothalamic-pituitary-adrenal (HPA) axis indexed by cortisol levels (Grunau et al., 2013; Brummelte et al., 2015), cognitive function (Grunau et al., 2009), and anxiety/depressive behaviors (Ranger et al., 2014).

Changes in maturation of the limbic system (Kinney, 2009) and basal ganglia (Peterson et al., 2000; Brunnemann et al., 2013; Scheinost et al., 2016) have been reported in children born preterm, associated with poorer behavioral (Rogers et al., 2017) and cognitive outcomes (Ball et al., 2015). Subcortical brain structures including hippocampus, amygdala, thalamus, and basal ganglia comprise the limbic system, involved in emotion, learning, and memory. Moreover, the thalamus is a relay center, directing sensory information from the periphery to other brain regions for processing, including the cerebral cortex. We recently found in infants born very preterm that thalamic volume, metabolism and structural maturation, were related to greater exposure to neonatal pain-related stress (Duerden et al., 2018; Schneider et al., 2018), and in turn, associated with poorer cognitive and motor outcomes (Duerden et al., 2018). However, the relationship between neonatal pain/stress exposure and the structures adjacent to thalamus, including the limbic system and basal ganglia, have not been examined.

There is wide variation in long-term outcomes of children born very preterm, even among those of similar gestational age, exposure to neonatal pain/stress and other clinical factors. Genetic vulnerability to early adversity has been identified, for example catechol-*O*-transferase (*COMT*) for pain sensitivity (Diatchenko et al., 2005; Hoth et al., 2006; Nackley et al., 2006), serotonin transporter [*SLC6A4*] in stress regulation (Lucki, 1998; Lesch, 2007) and brain-derived neurotrophic factor (*BDNF*) for brain synaptic plasticity (Gerritsen et al., 2012; Lakshminarasimhan and Chattarji, 2012; Kim et al., 2013). The Catechol-*O*-methyltransferase (*COMT*) gene encodes a key enzyme in the degradation of catecholamines (serotonin, dopamine, norepinephrine) (Lotta et al., 1995). *COMT Val158Met* is a common haplotype of the human *COMT* gene. The Met/Met genotype of the *COMT Val158Met* variant is associated with more than a three- to fourfold decrease in COMT enzyme activity and dopamine (DA) catabolism, which leads to an increase in DA availability in the prefrontal cortex (PFC) (Lotta et al., 1995; Chen et al., 2004). *COMT* Val158 leads to lower synaptic dopamine levels and poorer prefrontal functions with increased risk of mood disorders. On the other hand, the Met158 allele has been linked to greater pain sensitivity (Diatchenko et al., 2005; Nackley et al., 2006), and anxiety (Olsson et al., 2007).

Serotonin (5-HT) receptors are widely distributed in the brain, including regions regulating emotion, attention, cognition, and learning (Lucki, 1998; Lesch, 2007). Allelic variations in the serotonin transporter promoter region (*5HTTLPR*) influence gene transcription and serotonin transporter (SLC6A4) levels, and therefore have a critical role in determining intrasynaptic 5-HT levels. Alterations in 5-HT neurotransmission have been associated with reduced gray matter in areas of the limbic system (Pezawas et al., 2005). Finally, brain-derived neurotrophic factor (BDNF), an important neurotrophin widely expressed in the brain, especially in hippocampal regions, affects long-term neuronal survival, development and synaptic plasticity. Human postmortem (Chen et al., 2001) and animal studies have found that stress exposure modifies BDNF expression: decreased expression in hippocampus and increased in amygdala (Rasmusson et al., 2002; Govindarajan et al., 2006; Lakshminarasimhan and Chattarji, 2012). The *BDNF* Val66Met (rs6265) variant affects intracellular processing and secretion of BDNF (Egan et al., 2001). The Met allele encodes a precursor protein with impaired function which results in lower BDNF availability, and hence is associated with alterations of human hippocampal function and episodic memory (Egan et al., 2003).

In a targeted approach, we previously found gene polymorphisms associated with increased vulnerability to pain/stress exposure in very preterm neonates. We examined gene by environment interactions between neonatal pain/stress and genetic variations related to dopaminergic and serotonergic pathways, and also examined epigenetic changes in methylation in the promotor region of *SLC6A4* (Chau et al., 2014). We found that neonatal pain/stress exposure interacted with the *COMT* genotype to predict the level of *SLC6A4* methylation at age 7 years in children born very preterm, which in turn was associated with behavior. A group in Italy also reported similar findings, that exposure to neonatal pain/stress was associated with DNA methylation of *SLC6A4* in very preterm infants at term-equivalent age (Provenzi et al., 2015). Taken together, these studies suggest that long-term alterations in gene expression in infants born very preterm are at least partially induced by exposure to pain/stress of invasive procedures in the NICU. Moreover, we recently reported that the *BDNF* genotype moderated the association between neonatal pain/stress and cortisol (levels and reactivity) at age 7 years in boys, but not girls, born very preterm. Cortisol reactivity was associated with cognitive function and visual-motor integration in these children (Chau et al., 2017).

In the present study, we examined in school-age children born very preterm whether the extent of exposure to procedural pain/stress in the NICU, acting directly and/or through genetic interaction with *COMT, BDNF, 5HTTLPR*, is associated with development of subcortical structures in the limbic system (hippocampal network, amygdala and thalamus) and the basal ganglia (striatum, and globus pallidus), after accounting for clinical factors associated with prematurity. We hypothesize that: (1) neonatal pain/stress will be related to subregional volumes in the limbic system and basal ganglia; (2) genotypes will moderate the relationship between pain/stress and subcortical volumes; (3) relationships between pain/stress, genotypes, and outcomes will vary by subcortical region (e.g., hippocampus for working memory, amygdala for depressive/anxiety symptoms, and thalamus for visual motor integration). The findings of this study may inform possible pathways and vulnerability for pain-related stress to affect outcomes in children born very preterm.

## Materials and Methods

### Study Design and Participants

Participants were part of a larger longitudinal study of long-term effects of neonatal pain-related stress on neurodevelopment of children born very preterm (24–32 weeks gestation), e.g., (Grunau et al., 2007, 2009) admitted to the level III NICU at British Columbia’s Women’s Hospital between 2000 and 2004. Of 106 very preterm children seen at 8 years, 61 underwent MRI. Of these 61 children, two with periventricular leukomalacia (PVL) and/or intraventricular hemorrhage (IVH) grade 3 or 4 on neonatal ultrasound, confirmed on MR scans at 8 years, were excluded. Three children with ventriculomegaly and six who showed minimal to moderate white matter injury (≤3 lesions) on MR scans at school age were included in the study, since none of them were outliers on total brain and hippocampal volumes compared to the rest of the sample. An additional two children were excluded due to excessive movement artifact, therefore a total of 57 children (45% boys) comprised the study sample. All children in the final sample of 57 had a full scale IQ above 72 on the Wechsler Intelligence Scale for Children 4th Ed (WISC-IV) (Wechsler, 2003), and none had a major sensory or motor impairment.

The study was approved by the Clinical Research Ethics Board of the University of British Columbia and the British Columbia Children’s and Women’s Research Ethics Board. Written informed consent was obtained from parents and assent from children.

### Procedures

#### Clinical Data Collection

Medical and nursing chart review of neonatal data from birth to term equivalent was carried out by highly trained neonatal research nurses. Data collected included, but was not limited to, birth weight, gestational age (GA), small for gestational age (SGA; birth weight <10% tile), number of days on mechanical ventilation and/or oscillation, illness severity on day 1 [Score for Neonatal Acute Physiology (SNAP)- II (Richardson et al., 2001)], number of surgeries, presence of culture proven infection, and cumulative dose of morphine. The cumulative dose of morphine was calculated (intravenous dose plus converted oral dose) as the average daily dose adjusted for daily body weight, multiplied by the number of days the drug was given, as we have used previously (Grunau et al., 2009; Brummelte et al., 2012). We quantified exposure to neonatal invasive procedures as the number of skin-breaking procedures (e.g., heel lance, peripheral intravenous or central line insertion, chest-tube insertion, tape removal, and nasogastric tube insertion) during the stay in the NICU, as previously used (Grunau et al., 2005, 2009; Brummelte et al., 2012). Each attempt at a procedure was counted as one skin-break; all nursing staff in our NICU have been trained to precisely record each attempt.

#### Magnetic Resonance Imaging

The MRI was performed using a standard 12 channel head coil on a Siemens 1.5 Tesla Avanto (Berlin, Germany) with VB 16 software. The following images were acquired: a 3D T1 weighted SPGR sequence 18/9.2/256/1 mm/0/256 × 256 (TR/TE/FOV/Thickness/Gap/Matrix), axial FSE T2 4030/90/ 220/3 mm/0.1 mm/512 × 354, axial FLAIR 8900/87/5 mm/1 mm/ 256 × 154 and a 12 direction DTI sequence 7800/82/256/2 mm/ 0/128 × 128 using B values of 700 and 1000. All imaging sessions were performed without sedation. On the study day, each child first had a session in a mock scanner to acclimatize to the noise and feeling of undergoing MRI, followed by the actual study scan. Children were instructed to remain still and watched a video during the sessions that lasted approximately 30 min.

An experienced pediatric neuroradiologist (KP), blinded to the child’s medical history, assessed the MR scans for ventriculomegaly, cerebellar hemorrhage and severity of white matter injury, as previously described (Vinall et al., 2013). No child had a severe brain injury at school age [i.e., no cerebellar hemorrhage or severe white matter injury (i.e., >3 lesions or 2 with 5% hemisphere involved)].

#### MR Image Segmentation and Analysis

An automatic segmentation protocol based on the MAGeT Brain (Multiple Automatically Generated Templates) algorithm (Pipitone et al., 2014) was used to delineate the following: hippocampal subfields [cornus ammonis (CA) 1, subiculum, CA4/dentate gyrus (DG), CA2/CA3, stratum radiatum/lacunosum/molecular] (Winterburn et al., 2013), hippocampal white matter (alveus, fimbria, fornix, and mammillary bodies) (Amaral et al., 2018), basal ganglia (striatum and globus pallidus) and thalamus (Chakravarty et al., 2013) of preterm children (Guo et al., 2015). Total of 26 bilateral regions (13 regions in each hemisphere) were segmented and included in this study. All automated segmentations were visually inspected by an expert rater before inclusion (MP; RA).

#### Genotyping

Genomic DNA was extracted from neonatal whole blood samples using the Flexigene DNA Blood Kit (Qiagen, Valencia, California). The COMT Val158Met (rs4680) variant and BDNF Val66Met (rs6265) were genotyped using TaqMan SNP Genotyping Assay reagents and an Applied Biosystems 7300 Real Time PCR System (Applied Biosystems, Carlsbad, CA, United States). Call rate was 100%. Due to the low frequencies of the BDNF Met allele, subjects with the Met/Met and the Val/Met genotypes were grouped together in all analyses. The Hardy–Weinberg equilibrium of allelic distribution of the cohort was examined and compared to the population distribution by chi-square test.

The S and L alleles of SLC6A4 HTTLPR were identified as previously described in Lesch et al. (1996). Polymerase chain reaction was performed with oligonucleotide primers flanking the polymorphism (corresponding to nucleotide positions -1416 to -1397 [stpr5, 5_-GGCGTTGC CGCTCTGAATGC] and -910 to -888 [stpr3, 5_-GAGG GACTGAGCTGGACAACCAC]) of the 5_-flanking regulatory region of SLC6A4 to generate a 484-bp (S short allele) or a 528-bp (L long allele) polymerase chain reaction product. Polymerase chain reaction amplification was performed in a final volume of 30 μL with 50 ng of genomic DNA, 2.5 mM deoxyribonucleotides (dGTP/7-deaza-2_-dGTP = l/l), 0.1 μg of sense and antisense primers, 10 mM Tris hydrochloride (pH 8.3), 50 mM potassium chloride, 1.5 mM magnesium chloride, and 1 U of Taq DNA polymerase. For quality control, 5% of the samples were randomly chosen to be retested and their genotypes were consistent with previous results.

#### Cognitive, Motor and Behavioral Outcomes Measures

Children’s cognitive performance was assessed by Wechsler Intelligence Scale for Children 4th Ed (WISC-IV) composite scores [Verbal Comprehension Index (VCI), Perceptual Reasoning Index (PRI), Working Memory (WMI), Processing Speed Index (PSI)] (Wechsler, 2003).

Parents rated their child’s behavior using the Child Behavioral Check List (CBCL) for children ages 6–18 years (Achenbach and Rescorla, 2000). The internalizing and externalizing subscales *T*-scores were used as the indicator of child behavior in the current study. The internalizing scale encompasses anxious/depressed, withdrawn/depressed, somatic problems, whereas the externalizing scale includes aggressive and rule-breaking behaviors. Behavior Rating Inventory of Executive Function (BRIEF) Global Executive Composite (GEC) score was used to assess executive function behavior (Gioia et al., 2000). The Beery Developmental Test of Visual-Motor Integration (VMI) was used to measure visual-motor coordination (Beery and Beery, 2004); the Beery Motor Coordination and Visual Perception subscales were of particular interest for this study.

### Statistical Analysis

Constrained principal component analysis (CPCA) with interaction terms was used to examine the effects of seven neonatal clinical variables of interest (GA, neonatal infection, number of invasive procedures, number of surgeries, days on mechanical ventilation, illness severity on day 1, morphine exposure), *BDNF, SLC6A4*, and *COMT* genotypes, on the volumes of hippocampal subregions, hippocampus-related tracts, basal ganglia, thalamus and amygdala.

Constrained principal component analysis is a 2-step process, referred to as the external and internal analysis. First, the external analysis consists of a multivariate least squares multiple regression of the dependent measures on the independent measures, producing predicted and residual scores for each dependent measure. In the present study, the matrix of predicted scores reflect the variation in volume of hippocampus subregions and white matter tracts, amygdala, basal ganglia, and thalamus, in relation to the seven neonatal clinical variables, total brain volume and three genotypes, then by the interactions between clinical variables and genotypes. The major allele for each gene (e.g., *BDNF* Val66Met Val/Val) was the reference category in all analyses that included genotypes.

The second step, the internal analysis, comprised principal component analyses on each of the aforementioned matrices, including main effects and interaction terms. The resulting component solutions (overall, predicted, and residual solutions) were examined to determine which subregional volumes of the limbic system and basal ganglia could be explained by the neonatal clinical variables, genotypes, and interaction term. CPCA results were bootstrapped 1,000 times to compute confident intervals and *p*-values. Multiple comparison were adjusted by 5% Benjamini–Hochberg False discovery rate (FDR) (Benjamini and Hochberg, 1995) and Bonferroni method. Details of CPCA have been described previously (Ranger et al., 2013, 2015). Computations for CPCA were done using MATLAB v 8.5.0 (R2015a) (The MathWorks, 2010, Natick, MA, United States).

Given our interest in the functions supported by the subcortical structures comprising the limbic system and basal ganglia (e.g., memory and behavior), CPCA component loadings were examined for associations with WISC IV composite scores (VCI, PRI, WMI, and PSI), CBCL *T*-scores (Internalizing and Externalizing), BRIEF GEC scores, as well as the Beery VMI, Visual Perception and Motor coordination scores.

## Results

### Neonatal Clinical Factors Predict Limbic and Basal Ganglia Volumes at 8 Years

Participant characteristics are presented in Table 1. Genotype distribution is summarized in Table 2. The allelic distribution of our sample did not differ from the Hardy–Weinberg Equilibrium from the population.

**Table 1:** Participant characteristics.

Neonatal	*N* = 57 (26 boys, 31 girls)
Gestational age at birth (GA, wks)	29.7 (27.6–31.7)
Birth weight (g)	1285 (920–1560)
Small for gestational age (*n*, %)	6 (11)
Illness severity day 1 (SNAP-II)	8 (0–18.5)
Pain/stress (# invasive procedures)	74 (46.5–131)
Infants mechanically ventilated (*n*, %)	35 (61.4)
Days of mechanical ventilation	9 (0–71)
Culture proven infection (*n*, %)	14 (24.6)
Surgery (*n*, %)	11 (19.3)
Morphine exposure (mg/kg^I^)	0.03 (0–0.7)
Postnatal corticosteroid exposure (*n*, days)	6 (2–24)
**Child**	**Mean (range)**
Age at scan (years)	7.9 (7.7–8)
Total brain volume (cm^3^)	1397.0 (1123.4–1759.0)
Total right/left hippocampal volume (cm^3^)	214 (199–234)/221 (201–236)
Total right/left amygdala volume	111 (108–123)/117 (109–125)
Total right/left thalamus volume	623 (582–668)/588 (551–629)
WISC-IV Full Scale IQ	101 (73–135)
WISC-IV VCI	100 (77–136)
WISC-IV PRI	104 (69–147)
WISC-IV WMI	99 (77–120)
WISC-IV PSI	98 (75–131)
CBCL Internalizing Problems	53 (34–80)
CBCL Externalizing Problems	48 (33–77)
BRIEF GEC	53 (35–86)
Beery Visual Motor Integration	94 (75–112)
Beery Visual Perception	105 (77–143)
Beery Motor Coordination	92 (60–113)

*WISC-IV, Wechsler Intelligence Scale for Children 4th Ed; VCI, Verbal Comprehension Index; PRI, Perceptual Reasoning Index; WMI, Working Memory Index; PSI, Processing Speed Index; CBCL, Child Behavior Checklist; BRIEF, Behavior Rating Inventory of Executive Function; GEC, Global Executive Composite; Beery VMI, Beery Visual Motor Integration. Values are means and ranges, unless otherwise specified. ^*I*^Cumulative daily dose adjusted for daily body weight multiplied by the number of days drug received*.

**Table 2:** Genotypes distribution.

Genes	Genotypes	*N* = 57	HWE *p*-value^a^
BDNF Val66Met rs6265	Val/Val	38	0.655
	Val/Met + Met/Met	19	
SLC6A4 5HTTLPR	L/L	14	0.802
	L/S + S/S	43	
COMT Val158Met rs4680	Val/Val	17	0.956
	Val/Met	27	
	Met/Met	12	

*^*a*^p-values of independent 2-tailed t-test comparing Hardy–Weinberg Equilibrium (HWE) and study sample allelic distributions*.

The external analysis of CPCA showed that the predictor variables (7 neonatal clinical factors, total brain volumes, and *BDNF, SLC6A4, COMT* genotypes) accounted for 46.1% of the overall variance in volumes of hippocampal subregions, tracts, basal ganglia, thalamus and amygdala (Table 3). Two main components (PC1 and PC2) were extracted from the predicted solutions comprising neonatal clinical factors (NCF) and genotypes (G) independently, which corresponded, respectively, to 26.0 and 20.1% of the overall variance. Another component (PC NCFxG) reflected the interactions between the neonatal clinical factors and genotypes (NCF × G). All these extracted components (PC1, PC2, and PC NCFxG) were orthogonal (i.e., did not correlate with each other).

**Table 3:** Constrained principal component analysis (CPCA) results to explain variance in volumes of hippocampal subregions, tracts, basal ganglia, thalamus and amygdala in relation to neonatal clinical factors (NCF) and genotypes (G).

CPCA clinical and genotype variables	Overall	PC1	PC2	PC1 + PC2
Total variance	26.46	9.45	8.19	17.64
% variance	100.00	35.72	30.93	66.65
Variance explained by clinical variables and genotypes	13.79	6.87	5.32	12.19
% variance explained by clinical variables and genotypes	52.16	25.96	20.11	46.07
**CPCA clinical × genotype interaction**		**PC NCFxG**		
Variance explained by clinical variables **×** genotypes interaction	4.72	1.34	–	1.34
% Variance explained by clinical variables **×** genotypes interaction	17.84	5.08	–	5.08

*Each component accounts for a portion of the total variance: the first component accounts for the largest amount of variance, with each successive component accounting for a smaller amount of the total variance. PC1, principal component 1; PC2, principal component 2; PC1 + PC2, sum of PC1 and PC2; PC NFCxG, interaction term between neonatal clinical factors and genotypes*.

Component loadings, their bootstrapped *p*-values and FDR adjusted *p*-values for each hippocampal subregions, tracts, thalamus and amygdala volumes predicted by NCF, G independently and NCFxG interaction are summarized in Table 4. For the volumes explained by NCF and G independently, both components PC1 and PC2 negatively loaded on all hippocampal subregions, tracts, amygdala and thalamus volumes (i.e., indicating smaller volumes). The dominant loadings on the first component (PC1) were distributed bilaterally (on right and left hemispheres) on most of the subregions, but prominently (greater loadings) on amygdala and thalamus. For the second component (PC2), hippocampal subregions had the largest loadings. For the volumes explained by the NCFxG interaction, all subregions significantly explained by component PC NCFxG were negatively loaded and the dominant loadings were distributed mainly on the right hemisphere’s hippocampal subregions and related tracts.

**Table 4:** CPCA component loadings predicted by neonatal clinical factors (NCF) and genotypes (G) independently and interactively.

	PC1			PC2			PC NCFxG		
Brain Regions	Loadings	*p*-value^a^	FDR^b^	Loadings	*p*-value^a^	FDR^b^	Loadings	*p*-value^a^	FDR^b^
L_CA1	-0.259	0.093	0.105	-0.950	0.0000	0.000	-0.751	0.060	0.156
L_subiculum	-0.510	0.042	0.049	-0.736	0.0120	0.017	-0.130	0.758	0.984
L_CA4DG	-0.764	0.000	0.000	-0.562	0.0004	0.001	-0.738	0.018	0.058
L_CA2CA3	-0.521	0.166	0.180	-0.610	0.2175	0.226	-0.556	0.052	0.150
L_stratum	-0.432	0.006	0.009	-0.860	0.0000	0.000	-0.815	0.009	0.036
R_CA1	-0.427	0.005	0.009	-0.859	0.0001	0.000	-0.909	0.009	0.036
R_subiculum	-0.634	0.001	0.001	-0.720	0.0004	0.001	-0.254	0.476	0.858
R_CA4DG	-0.726	0.000	0.000	-0.646	0.0002	0.000	-0.802	0.000	0.003
R_CA2CA3	-0.584	0.033	0.040	-0.664	0.0283	0.037	-0.607	0.009	0.036
R_stratum	-0.425	0.007	0.010	-0.887	0.0000	0.000	-0.829	0.010	0.036
L_Alveus	-0.570	0.012	0.016	-0.739	0.0116	0.017	-0.773	0.006	0.036
L_Fimbria	-0.850	0.000	0.000	-0.361	0.1050	0.114	-0.257	0.495	0.858
L_Fornix	-0.642	0.000	0.000	-0.649	0.0001	0.000	-0.029	0.970	0.984
L_Mam	-0.350	0.469	0.469	-0.443	0.4073	0.407	0.282	0.384	0.832
R_Alveus	-0.552	0.026	0.034	-0.663	0.0128	0.018	-0.819	0.000	0.003
R_Fimbria	-0.800	0.000	0.000	-0.475	0.0054	0.009	-0.277	0.460	0.858
R_Fornix	-0.708	0.000	0.000	-0.651	0.0000	0.000	-0.457	0.379	0.832
R_Mam	-0.456	0.206	0.214	-0.684	0.0590	0.067	0.181	0.588	0.899
L_amygdala	-0.761	0.000	0.000	-0.611	0.0000	0.000	-0.326	0.534	0.867
R_amygdala	-0.820	0.000	0.000	-0.516	0.0001	0.000	0.217	0.718	0.982
L_striatum	-0.824	0.000	0.000	-0.484	0.0005	0.001	0.217	0.817	0.984
L_gp	-0.895	0.000	0.000	-0.363	0.0350	0.043	0.263	0.644	0.930
L_thalamus	-0.832	0.000	0.000	-0.475	0.0034	0.006	-0.036	0.953	0.984
R_striatum	-0.811	0.000	0.000	-0.505	0.0003	0.001	0.123	0.890	0.984
R_gp	-0.905	0.000	0.000	-0.357	0.0381	0.045	0.043	0.944	0.984
R_thalamus	-0.831	0.000	0.000	-0.467	0.0061	0.010	-0.011	0.984	0.984

*Negative loadings for each component indicate smaller regional volumes. The bigger the negative loading, the smaller the regional volume. PC1 principal component 1; PC2, principal component 2; PC NCFxG, principal component of the interaction term between neonatal clinical factors and genotypes; L, left; R, right. Left and right hippocampal subfields: cornus ammonis (CA) 1, subiculum, CA4/dentate gyrus (DG), CA2/CA3, stratum radiatum/lacunosum/molecular; Left and right hippocampal white matter: alveus, fimbria, fornix, and mammillary bodies (Mam); Left and right amygdala; Left and right basal ganglia: striatum, globus pallidus (gp) and thalamus. ^*a*^Red font indicates p < 0.0045 after Bonferroni adjustment for multiple comparisons. ^*b*^Red font indicates p < 0.05 after Benjamini–Hochberg false discovery rate (FDR) 5% overall error rate adjustment*.

Component 1 (PC1) and 2 (PC2) were negatively related to regional volumes of the limbic system (see Table 4). We then examined how each neonatal variable loaded on the components (Table 5). To understand the relationships between neonatal factors and regional brain volumes, Tables 4 and 5 need to be considered together. As expected, total brain volume was negatively loaded on both components (Table 5), indicating that bigger total brain volume was associated with larger volume of subregions (Table 4). Component 1 (PC1) reflecting more neonatal invasive procedures and more days of ventilation (Table 5), was related to smaller volumes particularly in amygdala and thalamus (Table 4); lower GA (Table 5) was associated with smaller volumes as well (Table 4). Component 2 (PC2) represented total number of surgeries (Table 5), where greater number of surgeries was associated with smaller volumes, particularly in the hippocampal subregions (Table 4). None of the genotypes significantly loaded on either components, indicating that on their own, genotypes were not associated with hippocampal subregions, tracts, amygdala and thalamus volumes.

**Table 5:** Predictor loadings for neonatal clinical factors and genotypes.

	PC1			PC2		
Variables	Loadings	*p*-value^a^	FDR^b^	Loadings	*p*-value^a^	FDR^b^
**Neonatal clinical factors**						
Infection	0.320	0.0126	0.023	0.105	0.4359	0.533
Invasive procedures	0.483	0.0001	0.000	-0.125	0.3465	0.533
Morphine	0.238	0.0620	0.097	0.265	0.0532	0.146
Surgery	-0.085	0.5011	0.612	0.419	0.0019	0.010
Mechanical ventilation	0.502	0.0001	0.000	0.152	0.2663	0.488
SNAP-II Day 1	0.333	0.0111	0.023	0.274	0.0367	0.135
GA	-0.460	0.0005	0.001	-0.106	0.4243	0.533
Total brain volume	-0.771	0.0000	0.000	-0.513	0.0001	0.001
**Genotypes**						
BDNF	-0.075	0.5743	0.632	0.051	0.7024	0.702
5HTTLPR	0.137	0.3232	0.444	-0.229	0.0860	0.189
COMT	-0.051	0.7057	0.706	-0.069	0.6166	0.678

*Both PC1 and PC2 were negatively related to regional volumes of the limbic system (Table 4). Significant positive loadings in this table indicate the relative contribution of each clinical factor and genotype for each component. For example, taking Tables 4 and 5 together, for PC1, more neonatal invasive procedures and more days on mechanical ventilation were related to smaller regional volume of CA4DG. PC1, principal component 1; PC2, principal component 2; Infection, neonatal culture proven infection; Morphine, morphine exposure, Surgery, number of surgeries; Ventilation, days on mechanical ventilation, SNAP-II day 1, score for neonatal acute physiology-II (i.e., illness severity on day 1); GA, gestation age. ^*a*^Red font indicate p < 0.0045 after Bonferroni adjustment for multiple comparisons. ^*b*^Red font indicate p < 0.05 after Benjamini Hochberg false discovery rate (FDR) 5% overall error rate adjustment*.

### Gene by Environment Interaction to Predict Subcortical Volumes

**FIGURE 1:**
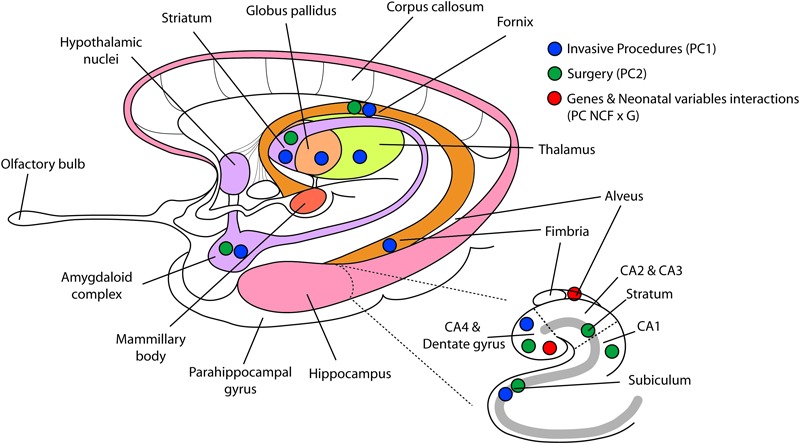
Reduced regional volumes in limbic system, basal ganglia, and thalamus. This figure displays the results of the constrained principal component analysis. Blue dots representing number of invasive procedures (component 1) are displayed on the brain regions that were significantly altered. Green dots representing more surgeries (component 2) are displayed on brain regions that were bilaterally reduced by the number of neonatal surgeries. Red dots, representing the interaction term between neonatal clinical factors and genotypes, show the two brain regions significantly related to this interaction term. PC1, principal component 1; PC2, principal component 2; PC NCFxG, principal component of the interaction term between neonatal clinical factors and genotypes.

We further examined how the interaction between each of the seven neonatal clinical factors, total brain volume and each of the three genotypes loaded onto the interaction term (PC NCFxG) (Table 6). In our statistical model all minor alleles were coded as 1 and the major alleles were 0, consequently negative loadings in the interaction terms represented smaller volumes with the minor alleles. Here we found that for very preterm children with the *COMT 158Met/Met* minor allele, greater neonatal invasive procedures exposure predicted smaller right hippocampal volumes (*p* = 0.0001); while greater GA was associated with larger right hippocampal volumes (*p* = 0.0001). For children with a *BDNF 66Met* major allele compared to those with *BDNF 66ValVal* genotype, presence of neonatal infection predicted larger right hippocampal subregional volumes (*p* = 0.0012). However, in those same children with a *BDNF 66Met* allele, greater number of surgeries was associated with smaller right hippocampal subregional volumes (*p* = 0.0002). Refer to Figure 1 for a comprehensive diagram of our CPCA model and findings.

**Table 6:** Predictor loadings for the interactions between each neonatal clinical factor (NCF) × genotype (G).

NCF		*G*	PC NCFxG		
Variables		Variables	Loadings	*p*-values^a^	FDR^b^
Infection	×	BDNF	-0.419	0.0012	0.007
Infection	×	SLC6A4	-0.261	0.0506	0.203
Infection	×	COMT	0.253	0.0615	0.211
Inv.procedures	×	BDNF	-0.012	0.9279	0.928
Inv.procedures	×	SLC6A4	0.039	0.7734	0.844
Inv.procedures	×	COMT	0.542	0.0001	0.001
Morphine	×	BDNF	0.282	0.0360	0.173
Morphine	×	SLC6A4	0.196	0.1388	0.278
Morphine	×	COMT	0.080	0.5504	0.695
Surgery	×	BDNF	0.500	0.0002	0.001
Surgery	×	SLC6A4	0.230	0.0742	0.223
Surgery	×	COMT	0.089	0.4995	0.686
Ventilation	×	BDNF	0.207	0.1272	0.278
Ventilation	×	SLC6A4	0.210	0.1170	0.278
Ventilation	×	COMT	0.203	0.1351	0.278
SNAP-II	×	BDNF	0.122	0.3741	0.599
SNAP-II	×	SLC6A4	0.089	0.5143	0.686
SNAP-II	×	COMT	0.177	0.1906	0.327
GA	×	BDNF	-0.018	0.8911	0.928
GA	×	SLC6A4	-0.059	0.6702	0.804
GA	×	COMT	-0.518	0.0001	0.001
Total brain vol	×	BDNF	0.105	0.4362	0.654
Total brain vol	×	SLC6A4	-0.043	0.7501	0.844
Total brain vol	×	COMT	0.187	0.1532	0.283

*The direction of the loadings here depends on the reference allele for each genotype, e.g., for Surgery × BDNF genotype, the reference was the major allele (Val) which was coded as 0, therefore more surgeries with the presence of the minor allele indicates greater loading on the component PC NCFxG. Taken together Tables 4 and 6, smaller CA4DG volume was related to more surgeries in the presence of the BDNF minor allele. NCF variables: Infection, neonatal culture proven infection; Inv.Procedures, number of invasive procedures; Morphine, morphine exposure, Surgery, number of surgeries; Ventilation, days on mechanical ventilation, SNAP-II, Score for Neonatal Acute Physiology-II on day 1 (i.e., illness severity on day 1); GA, gestational age. PC NCFxG, principal component of the interaction term between neonatal clinical factors and genotypes. ^*a*^Red font indicate p < 0.0045 after Bonferroni adjustment for multiple comparisons. ^*b*^Red font indicate p < 0.05 after Benjamini–Hochberg false discovery rate (FDR) 5% overall error rate adjustment*.

It must be noted that some very preterm children included in this study were exposed to clinically relevant medications, such as dexamethasone (*n* = 5), hydrocortisone (*n* = 2), fentanyl (*n* = 8), and midazolam (*n* = 6), whose intake has been associated with decreased volumes in subcortical structures (Tam et al., 2011; Ranger et al., 2015; Duerden et al., 2016). However, due to the small sample size within each of these categories, we could not include these clinical factors as covariates in our model. To ensure that our findings were not driven by these factors, we reanalyzed the data using case wise deletion of the affected subjects; results remained consistent with our initial findings.

### CPCA Components Correlations With Cognitive, Motor and Behavioral Outcomes

To examine whether alterations in subcortical brain volumes found in relation to neonatal clinical factors and genotypes in our sample of very preterm children were related to specific functional outcomes at 8 years, further analyses were conducted. CPCA component scores were extracted and correlated with WISC IV composite scores (VCI, PRI, WMI, and PSI), CBCL *T*-scores (Internalizing and Externalizing), BRIEF GEC scores, as well as the Beery VMI, Visual Perception and Motor coordination. Results are summarized in Table 7.

**Table 7:** Correlations between CPCA component scores and neurodevelopmental outcomes.

	PC1		PC2		PC NCFxG	
	*r*	*p*-values	*r*	*p*-values	*r*	*p*-values
WISC-IV VCI composite	-0.146	0.282	-0.0284	0.034	-0.147	0.280
WISC-IV PRI composite	-0.270	0.042	-0.232	0.082	-0.203	0.130
WISC-IV WMI composite	-0.142	0.298	-0.244	0.070	-0.363	0.006
WISC-IV PSI composite	-0.089	0.520	-0.088	0.522	-0.378	0.004
CBCL internalizing problems	-0.050	0.714	-0.254	0.056	0.040	0.767
CBCL externalizing problems	-0.070	0.607	-0.024	0.862	0.316	0.017
BRIEF GEC	0.041	0.764	0.042	0.754	0.303	0.022
Beery VMI	-0.056	0.678	-0.069	0.609	-0.050	0.713
Beery visual perception	-0.265	0.047	-0.250	0.061	-0.152	0.258
Beery motor coordination	-0.121	0.370	-0.332	0.012	-0.229	0.087

*The direction of the correlation depends on the measure, e.g., WISC-IV and Beery scores higher is better, whereas CBCL and BRIEF problem scores lower is better. For example, higher PC1 was significantly correlated with poorer WISC-IV Perceptual Reasoning and poorer Beery Visual Perception. Taking Tables 5 and 7 together, higher PC1 (more invasive procedures, more days on mechanical ventilation, lower GA, and smaller total brain volume) was related to poorer WISC-IV Perceptual Reasoning and poorer Beery Visual Perception. PC1, principal component 1; PC2, principal component 2; PC NCFxG, principal component of the interaction term between neonatal clinical factors and genotypes; WISC-IV, Wechsler Intelligence Scale for Children 4th Ed; VCI, Verbal Comprehension Index; PRI, Perceptual Reasoning Index; WMI, Working Memory; PSI, Processing Speed Index; CBCL, Child Behavior Checklist; BRIEF, Behavior Rating Inventory of Executive Function; GEC, Global Executive Composite. Significant p-values are in red font.*

The WISC IV Perceptual Reasoning Index (*r* = -0.27, *p* = 0.042) and Beery Visual Perception scores (*r* = -0.27, *p* = 0.047) negatively correlated with PC1, which was prominently loaded on the amygdala and thalamus (see Table 4), and mainly associated with neonatal invasive procedures (see Table 5). This suggested the possible vulnerability of amygdala and thalamus to neonatal invasive procedures, and poorer long-term outcomes on perceptual reasoning and visual perception. The WISC IV Verbal Comprehension Index (*r* = -0.28, *p* = 0.034) and the Beery Motor Coordination score (*r* = -0.33, *p* = 0.012) negatively correlated with PC2, which was prominently loaded on hippocampal subregions (see Table 4) and was associated with lower number of surgeries (see Table 5). Here, this surprising finding indicates a possible relationship between neonatal surgical procedures exposure and hippocampal-related structures, which in term alters executive functions at school age.

The interaction term (PC NCFxG) revealed to be the most strongly correlated with the neurobehavioral outcomes, indicating the interactions between genotypes (COMT, BDNF; see Table 6) and neonatal factors (infection, invasive procedures, surgery, GA; see Table 6), which were mainly loaded on CA4/DG and alveus. Here, this interaction term was associated with the WISC IV Working Memory (*r* = -0.36, *p* = 0.006) and Processing Speed scores (*r* = -0.38, *p* = 0.004), the CBCL externalizing behavior *T*-score (*r* = -0.32, *p* = 0.017), and the BRIEF GEC score (*r* = -0.30, *p* = 0.022). These findings indicate a potential vulnerability of the hippocampal structures and related tracks in very preterm children of a particular genotype, that were the sickest during their NICU admission (i.e., lower GA and higher exposure to invasive procedures, surgeries and infection).

## Discussion

This is the first study to integrate relationships among neonatal factors, subcortical brain structures and genetic moderators in relation to cognitive and behavioral outcomes at school age in children born very preterm. We found that greater exposure to neonatal pain/stress was associated with smaller regional volumes in the limbic system (hippocampal network, amygdala, and thalamus) and basal ganglia (striatum and globus pallidus) at age 8 years, after accounting for clinical factors associated with prematurity. *COMT* and *BDNF* genotypes moderated this relationship in both the hippocampal dentate gyrus and alveus. Moreover, smaller regional volumes in the limbic system and basal ganglia were associated with poorer cognitive performance and more behavioral problems, consistent with our hypotheses.

The hippocampal network consists of the hippocampal formation and its efferent pathways. The hippocampal formation is a subcortical structure, comprised of morphologically distinct but connected regions. After adjusting for total brain volume, our CPCA model indicated that among the seven neonatal clinical factors tested number of invasive procedures (indicator of neonatal pain exposure), days on mechanical ventilation and gestational age were associated with smaller regional volumes bilaterally of CA4DG, fimbria, fornix, amygdala, striatum, globus pallidus, thalamus, and unilaterally with right subiculum. This finding seem to lead to alterations in long-term neurodevelopmental outcomes, as shown by poorer perceptual reasoning and visual perception at 8 years. Many of these regions have been previously reported to be involved in spatial orientation and memory (Wible et al., 1992; Jessberger et al., 2009; Hescham et al., 2017) and our results are consistent with these findings. Given our previous findings of altered cortical and subcortical gray and white matter structures in relation to exposure to pain and other clinical factors related to prematurity in very preterm children (Ranger et al., 2013; Vinall et al., 2013; Ranger et al., 2015), it is not surprising to find in the present study such a wide-spread detrimental effect of this early-stress exposure on regional volumes of the limbic system and basal ganglia. Interestingly, these affected subregions are mostly interconnected with the thalamus, the relayer of sensory and motor signals. Recently, in an independent cohort of very preterm infants, we found that neonatal procedural pain-related stress was associated with poorer thalamic growth from early life to term equivalent age, in turn related to poorer cognition and motor function at 3 years corrected age (Duerden et al., 2018). This relationship between pain/stress and slower thalamocortical growth was also reported in a Swiss cohort of very preterm infants (Schneider et al., 2018). Taken together, these studies indicate the importance of early-life stress in relation to thalamocortical development.

Thalamocortical connectivity is undergoing rapid development during the late second trimester of fetal life and is critical for how sensory information, including pain, is transmitted throughout the brain and then processed (Kostovic and Judas, 2010). Connections between subplate and cortices are at maximal at 24–28 weeks gestation and are particularly vulnerable to excitotoxicity (Kostovic and Judas, 2010; Verriotis et al., 2016). Even a single heel lance in preterm neonates or one incision in immature rodent pups can induce a cascade of physiological, hormonal, inflammatory, as well as evoked electrophysiological (EEG) responses and hemodynamic changes in the brain (Fitzgerald and Beggs, 2001; Bartocci et al., 2006; Slater et al., 2010; Fabrizi et al., 2011; Worley et al., 2012; Goksan et al., 2015; Hartley et al., 2017). Thus, given that very preterm infants undergo on average about 10 invasive procedures daily, it is conceivable that cumulative *repeated* exposure to invasive procedures could impact several important interconnected networks during this critical period of brain maturation. We have previously shown that neonatal exposure to pain-related stress was associated with long-term alterations in the spectral architecture of spontaneous brain oscillations, suggesting changes in thalamocortical connectivity (Doesburg et al., 2013). The associations between neonatal pain-related stress and smaller regional volumes in the major hippocampal output pathway (subiculum, fimbria, and fornix), basal ganglia (globus pallidus and striatum) and thalamus found in this study extend our previous findings, and confirm our recent reports of associations between neonatal procedural pain/stress and altered functional connectivity between thalamus and sensorimotor cortices in very preterm infants (Duerden et al., 2018; Schneider et al., 2018). Findings from our present study provide more detailed locations for potential effects of pain-related stress in subcortical brain structures.

Another major finding in this study was that the number of neonatal surgeries was associated with smaller volumes bilaterally in the CA1, CA4DG, hippocampal stratum, fornix, amygdala, striatum, and unilaterally in the right subiculum and CA2CA3 regions. Surprisingly, thalamic volume was not associated with surgery. However, it is difficult for us to speculate on the role that surgery plays in explaining the regional brain volumes since we cannot account for type of surgery (e.g., major versus minor) or duration, due to our limited sample size. We have previously reported that the number of neonatal surgeries (controlling for other neonatal confounders and concurrent maternal factors) predicted more anxiety/depressive symptoms at 8 years in very preterm children (Ranger et al., 2014). Further studies in larger cohorts of very preterm children are needed to examine in more detail the role of location and duration of surgery, as well as type and dosing of analgesic and sedative medications administered.

It is interesting that only the right subiculum region in the hippocampus was associated with neonatal invasive procedures and number of surgeries. The subiculum receives input signals from CA1 and outputs signals to the entorhinal and prefrontal cortices. The subiculum is situated at a pivotal junction between the hippocampus and the entorhinal cortex, and is thought to be involved in mnemonic processing and spatial navigation (O’Mara et al., 2001). Given the association between right hippocampus activity and mood disorder such as depression and anxiety (Malykhin et al., 2010; Mathias et al., 2016), and the finding of overall asymmetry in subiculum, further examination of this particular structure remains an interesting target for future studies.

To investigate possible pathways involved in the long-term neurodevelopmental outcomes of children born prematurely, we examined the interactions between neonatal clinical factors and genetic variants that are related to the availability of neurotransmitters dopamine (COMT) and serotonin (SLC6A4), and neurotrophin (BDNF), involved in neurogenesis. Interestingly, genotypes alone did not predict subcortical volumes in our sample of very preterm children at school age. Only interactions between genotypes and certain neonatal clinical factors were related to volume of the hippocampal cornu ammonis 4/dentate gyrus (CA4DG) and alveus. Surprisingly, this gene by environment effect was only found for the *COMT* and *BDNF* genotypes, but not *SLC6A4.* Specifically, smaller right hemisphere CA4DG and alveus were predicted by greater exposure to neonatal invasive procedure in very preterm children with *COMT 158Met/Met*, and by neonatal infection in those with the minor allele of *BDNF Val66Met*. Children with *COMT 158Met/Met* genotype have been shown to have increased pain sensitivity (Zubieta et al., 2003; Diatchenko et al., 2005; Ahlers et al., 2013; Sadhasivam et al., 2014). An interactive effect on hippocampal volumes of early adversity and *BDNF* Met-carrier status has been described in major depressive disorder and psychosis (Aas et al., 2013; Carballedo et al., 2013). Moreover, hippocampus CA4DG has been shown to contribute to memory formation (e.g., Saxe et al., 2006; Jessberger et al., 2009; Thompson et al., 2014). The alveus, an important bundle of white matter fibers, contains axonal fibers from the DG and from pyramidal neurons of CA3, CA2, CA1 and subiculum to form the fimbria/fornix (reviewed by O’Mara et al., 2001). Early-life stress is associated with decreased neurogenesis and effects can persist throughout life, for example stress is one of the most potent inhibitors of neurogenesis in the hippocampus (Korosi et al., 2012). Hippocampal development is vulnerable to infection, hypoxia/ischemia, undernutrition, fetal intra-uterine growth retardation as well as stress (Lodygensky et al., 2008; Sizonenko et al., 2008), all of which are complications that are associated with preterm birth. In the rodent pup, neonatal stress and/or noxious stimulation is associated with long-term alterations in hippocampal volumes (Malheiros et al., 2013; Victoria et al., 2013; Lima et al., 2014) and short-term neurochemistry (Mooney-Leber et al., 2018). Consistent with the animal work, altered hippocampal morphology and reduced volumes have also been found in infants, children and adolescents born preterm compared to full-term peers (Nosarti et al., 2002; Thompson et al., 2008; Guo et al., 2015). Therefore, it appears that the hippocampus CA4DG region and the output pathway are particular sensitive to neonatal pain-related stress and infection exposure, which in turn contribute to the long-term cognitive and behavioral outcomes in children born very preterm (e.g., working memory, processing speed, and aggressive/rule-breaking behaviors).

Our findings that both postnatal infection and neonatal pain-related stress contributed to reduced volume in hippocampal subregions, suggests an important role of the immune system, given the close relationships between stress and immune function. The immune system plays a critical role in shaping the brain (reviewed by Bilbo and Schwarz, 2012), especially during the last fetal trimester and early postnatal life, which coincides with the timing of very preterm infant exposure to NICU care. In a neonatal rodent model, even a single invasive procedure induced immune responses, with effects evident until adulthood (Beggs et al., 2012). A growing body of evidence points to the early-life environment as central in shaping how the brain and immune system interact and develop, with significant consequences on behavioral outcomes across the lifespan (reviewed by Bilbo and Schwarz, 2009). Indeed, increasing our understanding of how an altered immune system may contribute to changes in brain and thereby later neurobehavioral outcomes, is an important area for future research in this population.

We recognize that this study has limitations, particularly the relatively limited sample size for examining interactions between neonatal clinical factors and genotypes. Unfortunately, due to the sample size, we could not examine sex differences. Nonetheless, unique to our study is the multi-modal approach of genotyping and neuroimaging in relation to neurobehavioral outcomes. Poorer cognitive function and behavior is still evident in adulthood in this vulnerable population (e.g., Grunau et al., 2004). Our work begins to unravel the complexity of pathways involved in outcomes of children born very preterm.

## Conclusion

In a sample of very preterm children assessed at school age, neonatal clinical factors independently predicted a wide-spread of subregional volumes in the limbic system and basal ganglia, suggesting potential neurophysiological pathways between specific neonatal factors and subcortical structures. Neonatal exposure to invasive procedures, days on mechanical ventilation, and GA predicted smaller volumes in the CA4DG, fimbra, fornix, amygdala, striatum, globus pallidus, thalamus, and subiculum, which in turn were related to poorer perceptual reasoning and visual perception. Alongside, we showed that the number of surgeries during NICU admission predicted volumes of CA1, CA2CA3, CA4DG, hippocampal stratum, fornix, amygdala, striatum, and subiculum, which revealed to be associated with poorer verbal comprehension and motor coordination. Finally, through a gene by environment interaction analysis, we demonstrated that *COMT* genotype interacted with neonatal invasive procedures, while *BDNF* genotype interacted with occurrence of neonatal infection to predict volume of CA4DG and alveus. This interaction was related to poorer working memory, processing speed, and executive functions, as well as more externalizing behaviors at 8 years in children born very preterm. Therefore, these important structures for emotional regulation, executive function and motor coordination appear to demonstrate differential vulnerability to early environmental adversity, which can be moderated by specific genotypes, and in turn are associated with altered child neurodevelopmental outcomes. These findings begin to address factors underlying the diversity of long-term outcomes in children born very preterm and the complexity that interplays when examining how exposure to early-life stress may impact brain development both at the structural and functional level.

## Data Availability

The datasets for this manuscript are not publicly available because privacy issues. Requests to access the datasets should be directed to rgrunau@bcchr.ca.

## Author Contributions

REG conceived and designed the study. CC conducted the genotyping. KP was key to MRI acquisition and read all scans. MP and RA performed MRI data processing and segmentation. CC conducted statistical analyses. CC, MR, RG, and SM interpreted the results. CC, MR, and RG drafted the manuscript. All authors edited and revised the manuscript and approved the final version of manuscript.

## Conflict of Interest Statement

The authors declare that the research was conducted in the absence of any commercial or financial relationships that could be construed as a potential conflict of interest.
